# Incidence, long-term predictors and progression of chronic kidney disease among African migrants and non-migrants: the transcontinental population-based prospective RODAM cohort study

**DOI:** 10.1136/bmjgh-2024-016786

**Published:** 2025-01-20

**Authors:** Muhulo Muhau Mungamba, Felix P Chilunga, Eva L van der Linden, Erik Beune, Engwa A Godwill, Charles F Hayfron-Benjamin, Karlijn Meeks, Samuel N Darko, Sampson Twumasi-Ankrah, Ellis Owusu-Dabo, Liffert Vogt, Bert-Jan H van den Born, Benedicta N Chungag, Charles Agyemang

**Affiliations:** 1 Public and Occupational Health, University of Amsterdam, Amsterdam, The Netherlands; 2 Human Biology, Walter Sisulu University, Mthatha, South Africa; 3 Department of Public and Occupational Health, University of Amsterdam, Amsterdam, The Netherlands; 4 Department of Vascular Medicine, University of Amsterdam, Amsterdam, The Netherlands; 5 Department of Biological and Environmental Sciences, Walter Sisulu University, Mthatha, South Africa; 6 Department of Anaesthesia and Critical Care, Korle Bu Teaching Hospital, Accra, Ghana; 7 National Institutes of Health, Bethesda, Maryland, USA; 8 Department of Molecular Medicine, Kwame Nkrumah University of Science and Technology, Kumasi, Ghana; 9 Department of Statistics and Actuarial Science, Kwame Nkrumah University of Science and Technology, Kumasi, Ghana; 10 Kwame Nkrumah University of Science and Technology, Kumasi, Ghana; 11 Department of Internal Medicine, University of Amsterdam, Amsterdam, The Netherlands; 12 Department of Public and Occupational Health, Amsterdam Public Health Research Institute, Amsterdam, The Netherlands; 13 Division of Endocrinology, Diabetes, and Metabolism, Department of Medicine, Johns Hopkins University School of Medicine, Baltimore, Maryland, USA

**Keywords:** Global Health, Epidemiology, Public Health, Cardiovascular disease, Cohort study

## Abstract

**Background:**

Limited longitudinal data exist on chronic kidney disease (CKD) in African populations undergoing epidemiological transitions. We investigated incidence, long-term predictors and progression of CKD among Ghanaians residing in Ghana and Ghanaian migrants in the Netherlands (Amsterdam).

**Methods and findings:**

We analysed data from 2183 participants in the transcontinental population-based prospective Research on Obesity and Diabetes among African Migrants cohort, followed for approximately 7 years. CKD incidence and its progression to end-stage kidney disease (ESKD) were defined using Kidney Disease: Improving Global Outcomes (KDIGO) criteria. CKD incidence was calculated using age- and sex standardisation for those without CKD at baseline. Long-term predictors of CKD incidence were identified using one-step robust Poisson regression. CKD progression to ESKD from baseline was also assessed using robust Poisson regressions. Overall age- and sex standardised CKD incidence was 11.0% (95% CI 9.3% to 12.3%) in the population, with Ghanaians residing in Amsterdam at (7.6%; 5.7% to 9.5%) and Ghanaians residing in Ghana at (12.9%; 10.9% to 14.9%). Within Ghana, rural Ghanaians had similar CKD incidence to urban Ghanaians (12.5%; 8.5% to 15.5% vs 12.3%; 8.2% to 15.8%). Residence in Amsterdam was associated with lower CKD incidence compared with Ghana after adjustments (incidence rate ratio=0.32; 0.13–0.77). CKD incidence predictors were advanced age, female sex, alcohol consumption, uric acid levels and hypertension. CKD progression to ESKD was 2.3% among Ghanaians residing in Ghana and 0.0% among Ghanaians residing in Amsterdam.

**Conclusion:**

One-tenth of Ghanaians developed CKD over 7 years, with higher incidence in Ghana compared with Europe. Age, female sex, alcohol use, uric acid levels and hypertension were predictive factors. CKD progression to ESKD was minimal. High CKD incidence among Ghanaians, especially those residing in Ghana, calls for in-depth assessment of contributing factors and targeted interventions.

**WHAT IS ALREADY KNOWN ON THIS TOPIC**

- Chronic kidney disease (CKD) poses a significant global health challenge, with most longitudinal studies conducted in high-income countries. African populations, undergoing rapid urbanisation and migration, lack comprehensive longitudinal data, limiting understanding of CKD dynamics.

**WHAT THIS STUDY ADDS**

- Our 7-year study of 2183 Ghanaians residing in Ghana and Ghanaian migrants in the Netherlands reveals a high CKD incidence comparable to high-income countries. Contrary to expectations, Ghanaian migrants in the Netherlands had a lower CKD incidence despite higher cardiometabolic risks than their counterparts residing in Ghana. Key predictors of CKD include age, sex, alcohol consumption, uric acid levels and hypertension. Minimal progression to end-stage kidney disease (ESKD) was observed.

**HOW THIS STUDY MIGHT AFFECT RESEARCH, PRACTICE OR POLICY**

- The findings emphasise the need for detailed investigations into the high CKD incidence in Africa and the relatively low progression rates to ESKD, guiding the development of context-specific public health programmes. Understanding urbanisation, migration and epidemiological transitions is vital for developing effective public health programmes and policies.

## Introduction

 The African continent is currently undergoing rapid urbanisation, with a large number of people moving from rural to urban areas in search of better economic opportunities and living conditions.^1^ This urban transition brings about significant changes in lifestyle, including shifts in diet, physical activity and exposure to harmful environmental factors.^2 3^ These changes may contribute to the increasing prevalence of non-communicable diseases, including chronic kidney disease (CKD). Additionally, the migration of Africans to high-income regions like Europe has increased in recent years.^4^ Migrants face unique challenges related to nutrition, access to healthcare, cultural adaptation and socio-economic factors, all of which can influence their risk and progression of CKD.^5^

Several studies have shed light on the prevalence and risk factors of CKD in different African contexts, including the effects of urbanisation and international migration. For example, a systematic review in 2014 found a prevalence of 12% in urban areas and 17% in rural areas in the sub-Saharan Africa (SSA) region.^6^ A previous study from our group comparing Ghanaians living in rural and urban settings in Africa and those who migrated to Europe reported a prevalence of 13%, 14% and 10%, respectively.^7^ A recent study conducted in Uganda, South Africa and Malawi revealed national prevalence rates ranging from 10% to 20%.^2^ These studies have identified common risk factors such as advanced age, diabetes and hypertension for CKD in Africans, as well as some specific risk factors within the African context, like HIV infection and treatment, and use of herbal medications.^2^ Despite this extensive cross-sectional knowledge, there is a large knowledge gap concerning incidence, long-term predictors and progression of CKD among Africans undergoing epidemiological transitions.

While cross-sectional studies provide useful information on the overall disease burden in the population and associated risk factors, understanding the incidence of CKD and its long-term predictors is crucial for developing effective preventive strategies and targeted interventions. For example, tracking new cases and natural evolution of CKD can help identify changes in disease patterns and allocate resources and interventions more effectively. To date, the only four studies that have longitudinally studied CKD in African populations have been limited in scope, sample size or follow-up duration.^8 9^ For instance, a study on CKD progression in an urban hospital in South Africa followed 297 participants for 2 years only, potentially not capturing the full extent of CKD progression.^8^ Another study in urban Ghana on CKD progression only included HIV-positive adults, thereby limiting generalisability.^9^ Additionally, two clinically based studies in Ethiopia examined CKD incidence among patients with diabetes only also limiting generalisability.^10 11^ Moreover, none of these studies considered the influence of urbanisation, epidemiological transitions or international migration.

Therefore, this study aimed to address these gaps by conducting a prospective study for a comprehensive understanding of the incidence, long-term predictors and progression of CKD across the spectrum of epidemiological transitions: rural Ghana, urban Ghana and Ghanaian migrants in Europe. We hypothesised that Ghanaian migrants in Europe would have higher CKD incidence and progression due to higher levels of unhealthy lifestyle factors and comorbidities such as diabetes and hypertension, followed by urban Ghanaians, and rural Ghanaians.

## Methods

### Study design and population

The study used data from the transcontinental population-based Research on Obesity and Diabetes among African Migrants (RODAM) prospective cohort, which aims to examine complex gene-environmental interactions and their role in development of cardiometabolic diseases among African migrants and non-migrants. The cohort details have been previously published.^12^ In brief, a total of 4573 Ghanaian participants over 18 years were recruited and followed up in rural Ghana, urban Ghana and Amsterdam, the Netherlands. Baseline data were collected between 2012 and 2015, with follow-up spanning 2019–2021. In Ghana, urban and rural sites were selected purposively in the Ashanti region. Participants were randomly chosen from 30 enumeration areas, based on the 2010 census. In Amsterdam, Ghanaian participants were randomly selected from municipal register using country of birth indicators. Data collection, including demographics, lifestyle and cardiometabolic factors, followed a standardised approach, involving questionnaires, anthropometric measurements and venous blood samples across all locations.

### Chronic kidney disease

Participants provided early morning urine samples to analyse albumin and creatinine. Urinary albumin concentration (in mg/L) was determined using an immunochemical turbidimetric method and urinary creatinine concentration (in mmol/L) using kinetic spectrophotometric method (Roche Diagnostics). Albuminuria categories were based on urine albumin-to-creatinine ratio (ACR): A1 (<3 mg/mmol; normal to mildly increased), A2 (3–30 mg/mmol; moderately increased) and A3 (>30 mg/mmol; severely increased).^13^

Estimated glomerular filtration rate (eGFR) was assessed using the serum creatinine-based race-free CKD-EPI 2021 equation^14^. This equation was chosen because it provides better estimates of GFR in African populations compared with traditional methods, and due to the unavailability of iohexol measurements in our study, which is considered the best method for eGFR estimation in Africans.^2^ Serum creatinine concentration (mmol/L) was determined using a kinetic colorimetric spectrophotometric isotope dilution mass spectrometry calibrated method (Roche Diagnostics).

CKD was defined using KDIGO criteria: an eGFR < 60 mL/min/1.73 m² (stages 3a to 5) and/or ACR of ≥3 mg/mmol.^15^ For individuals falling within eGFR stages 1 and 2, albuminuria (ie, ACR ≥3 mg/mmol) was employed as an additional diagnostic criterion.

### Progression to end-stage kidney disease

CKD progression from baseline to end-stage kidney disease (ESKD) was assessed using KDIGO criteria^15 16^:

1. Progression to eGFR stage 5 (<15 mL/min/1.73 m²), necessitating chronic dialysis therapy or kidney transplantation.
2. A decline of 50% or more in eGFR from initial level.

### Other measurements

In addition to measurements related to CKD and its progression to ESKD, we collected a comprehensive set of measurements from the RODAM cohort for analysis. These include age, sex, education levels, tobacco smoking, dietary patterns, alcohol consumption, physical activity, anthropometrics, blood pressure (BP), fasting blood glucose, hypertension, diabetes and use of medications.

Age was recorded in years, and sex was categorised into male and female. Educational level was assessed based on the highest education attained, with categories including never been to school, lower vocational, intermediate vocational and higher vocational. Smoking status was categorised as yes, or no, while alcohol use in the last 12 months was categorised as yes or no. The WHO steps questionnaire was used to derive physical activity in metabolic equivalent (hours/week, which included physical activity at work, while commuting and in leisure time. Answers were subsequently classified based on the guidelines of the International Physical Activity Questionnaire (IPAQ) group into three levels of total physical activity (low, moderate, high).^17^,^19^ Dietary patterns were obtained from a standardised semiquantitative Ghana-specific food propensity questionnaire (Ghana-FPQ) that queried for the usual dietary intake of 134 food groups of the past 12 months and were categorised into mixed pattern, animal product pattern and roots, tubers and plantain pattern classifications.^19^

Body weight was measured in light clothing without shoes using SECA 877 scales with a precision of 0.1 kg. Height was measured without shoes using a portable stadiometer (SECA 217) with a precision of 0.1 cm. Body mass index (BMI) was calculated as weight divided by height squared (kg/m^2^). Overweight was defined as a BMI of between 25 and 30 kg/m^2^, while obesity was defined as a BMI of 30 kg/m^2^ or higher. Waist circumference was measured in centimetres at the midpoint between the lower rib and the upper margin of the iliac crest. All these anthropometric measures were taken twice, and the mean was used for analysis.

BP was measured three times while participants were in a seated position after resting for at least 5 min. The measurements were taken using the Microlife WatchBP Home, a validated semiautomated device. The mean of the last two BP measurements was used for analysis. Hypertension was defined as systolic BP of 140 mm Hg or higher and/or diastolic BP of 90 mm Hg or higher, or the use of antihypertensive medication per WHO criteria.^18^

Fasting plasma glucose concentration was determined using the enzymatic hexokinase method. Type 2 diabetes was defined based on the diagnostic criteria set by the WHO, including a fasting glucose level of 7.0 mmol/L or higher, current use of medication prescribed for diabetes treatment or self-reported physician-diagnosed diabetes.^20 21^ Serum uric acid concentration was measured using an enzymatic method (Trinder) in µmol/L. For total cholesterol (TC) and high-density lipoprotein cholesterol (HDL) concentration, a ready-to-use reagent for colorimetry was used. Additionally, low-density lipoprotein (LDL) was calculated using the Friedewald equation. All biochemical analyses were performed using ABX Pentra 400 chemistry analyse (ABX Pentra 400; Hiroba ABX, Darmstadt, Germany).

Medication use was assessed by asking participants to bring their prescribed medications to the physical exam, and these were subsequently coded and categorised. For hypertension, antihypertensive medications included were diuretics, β-blockers, ACE inhibitors or angiotensin receptor blockers, either individually or in combination. For diabetes, hypoglycaemic medications were considered, including metformin, sulfonylureas and insulin.

### Patient and public involvement

Community leaders played a central role in the design, recruitment and dissemination phases of the study. Identified through local organisations such as churches and mosques, and supported by key community figures, these leaders collaborated with researchers to develop protocols and recruitment strategies tailored to the community’s priorities and preferences. Public engagement was further strengthened through partnerships with media outlets (radio and television) and healthcare organisations. Information about the study was shared through local media, including radio and television, and formal notification letters were sent to selected health and community authorities. Team members actively engaged with the communities by staying on-site and organising mini clinics over a period of 1–2 weeks to encourage participation. Study findings were disseminated through seminars, community gatherings (durbars) and broadcasts on radio and television stations.

### Statistical analyses

Data analysis used R V.4.2.1 and was divided into two parts based on the presence or absence of CKD at baseline. For participants without CKD at baseline, we presented baseline characteristics: mean±SD for normally distributed data, median (IQR) for skewed data and frequencies/percentages for categorical data. Age- and sex-standardised CKD incidence proportions were estimated using direct standardisation (standardised to the structure of the total sample), by geographical location and metabolic factors (obesity, diabetes, hypertension). Long-term predictors of CKD incidence (sociodemographics, lifestyle factors, anthropometrics, metabolic measurements and comorbidities) were explored using one-step robust Poisson regression. Variance inflation factor (VIF) guided exclusion of collinear variables (VIF > 4). Associations between geographical location (predictor) and CKD incidence (outcome) were assessed using robust Poisson regression models sequentially adjusted for sociodemographics, lifestyle factors, anthropometrics, comorbidities and use of antihypertensive medications. We reported incidence rate ratios (IRRs) and 95% CIs. All tests were two-tailed, with an alpha level of 0.05.

Among participants with CKD at baseline, we also reported baseline characteristics, determined age- and sex-standardised proportions of CKD progression to ESKD, as well as associations between geographical location and CKD progression to ESKD using similar procedures as above.

### Sensitivity analyses

To investigate the potential bias from loss to follow-up, we conducted a sensitivity analysis. Given that a substantial proportion of the initial sample was lost, we compared the baseline characteristics of the participants who remained in the study with those who were lost to follow-up. This analysis aimed to test the validity and reliability of our findings by assessing any potential differences between these two groups. t-tests were used to test the difference between continuous variables (normally distributed), while χ^2^ tests were used to test the differences between categorical variables.

## Results

### Baseline characteristics of participants

**Figure 1:**
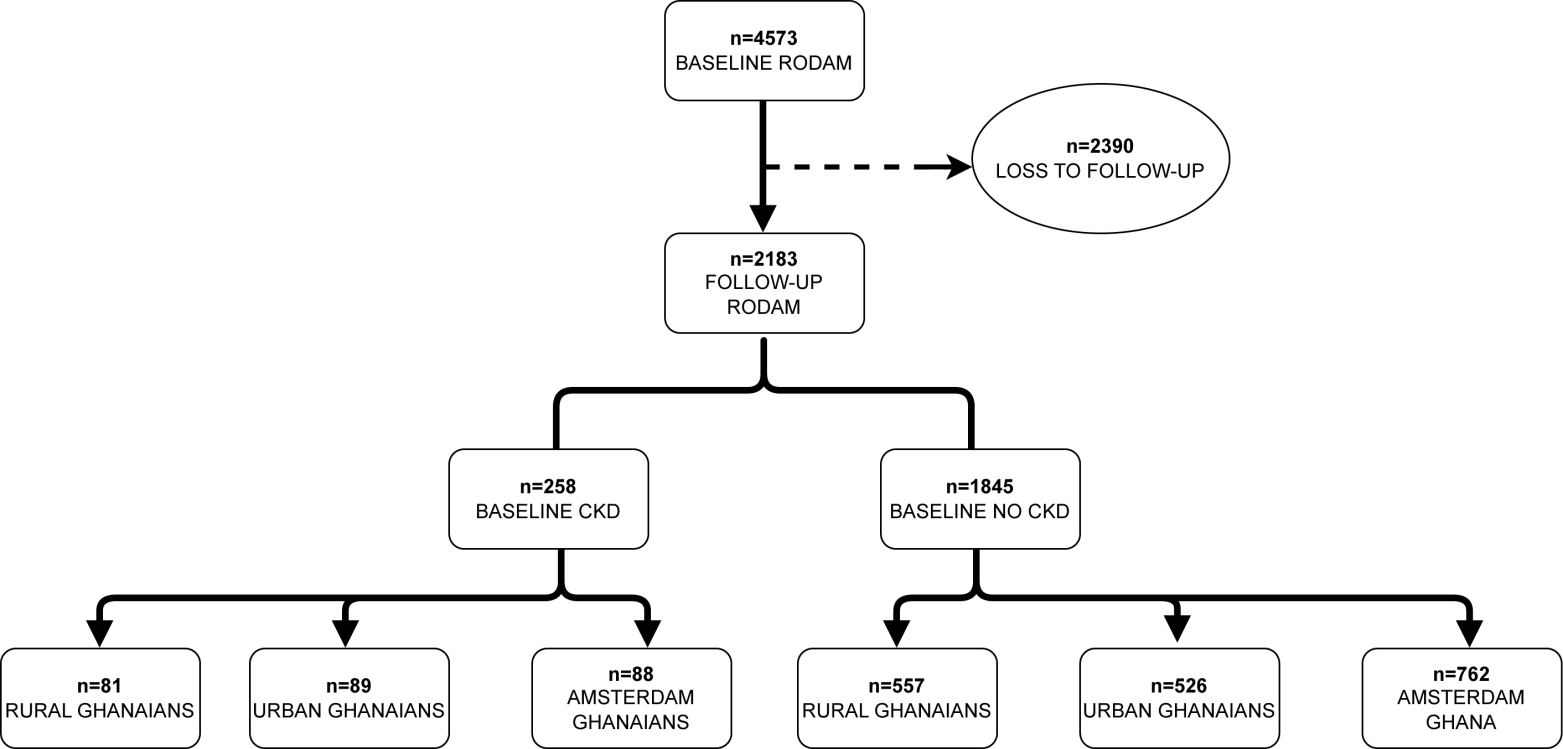
Flow diagrams of study participants. CKD, chronic kidney disease; RODAM, Research on Obesity and Diabetes among African Migrants.

Between 2015 and 2021, we followed 4573 RODAM participants. A total of 2390 (52%) were lost to follow-up, leaving 2183 (48%) participants for the present assessment (figure 1). Among these participants, 1845 did not have CKD at baseline, with rural Ghanaians accounting for 557 (30%), urban Ghanaians for 526 (29%) and Amsterdam Ghanaians for 762 (41%) participants. There were 258 participants with CKD at baseline, with 81 (31%) in rural Ghana, 89 (35%) in urban Ghana and 88 (34%) in Amsterdam (figure 1). All included participants had no CKD at baseline.

The majority (63%) of participants were women, with a mean age of 46 years (SD=11 years). Ghanaians in Amsterdam were more likely to have higher education levels and be employed full-time (46% vs 27.0% in Ghana). Regarding lifestyle, Ghanaians in Amsterdam were more likely to smoke and consuming a more varied diet. Anthropometric measures showed that they also had a higher prevalence of obesity (32.9%), with higher BMI and waist-to-hip ratios compared with those residing in Ghana. They also presented with higher HDL cholesterol, triglycerides, uric acid and eGFR. In terms of disease prevalence, Amsterdam Ghanaians had higher rates of hypertension (52.6% vs 28.6%) and diabetes (9.3% vs 4.4%), requiring more antihypertensive and antidiabetic medications (table 1). Within Ghana, urban residents were more likely to be highly educated but also had a more unhealthy lifestyle, higher obesity rates and more cardiometabolic comorbidities compared with their rural counterparts (online supplemental S1 table).

**Table 1:** Participants without chronic kidney disease at baseline: baseline characteristics of Amsterdam versus Ghana

Variables	Totaln=1845	Amsterdamn=762	Ghanan=1083	P value
*Demographics*				
Follow-up time (years), mean (SD)	6.7 (0.67)	6.78 (0.90)	6.68 (0.47)	<0.001
Age, mean (SD)	46 (10.9)	46 (9.6)	46 (12)	0.050
Sex, n (%)				
Females	1170 (63.4)	460 (60.4)	710 (65.6%)	0.023
Males	675 (36.6)	302 (39.6)	373 (34.4%)	
Education, n (%)				
Never/elementary	719 (41.0%)	217 (30.5%)	502 (48.2%)	<0.001
Lower secondary	666 (38.0)	264 (37.1)	402 (38.6%)	
Higher secondary	274 (15.6)	177 (24.9)	97 (9.3%)	
Tertiary	94 (5.4)	54 (7.6)	40 (3.8%)	
Employment status, n (%)				
Full time	445 (32.2)	172 (46.2)	273 (27.0%)	<0.001
Part-time	739 (53.5)	89 (23.9)	650 (64.4%)	
Social benefits	71 (5.1)	69 (18.5)	2 (0.2%)	
Retired	15 (1.1)	1 (0.3)	14 (1.4%)	
Full-time homemaker	17 (1.2)	6 (1.6)	11 (1.1%)	
Unable to work	83 (6.0)	30 (8.1)	53 (5.2%)	
Student	12 (0.9)	5 (1.3)	7 (0.7%)	
*Anthropometry information*				
Body mass index (kg/m^2^), median (IQR)	26.0 (22.4–29.7)	28.0 (25.2–30.8)	24.2 (20.7–28.3)	<0.001
Waist-hip ratio, median (IQR)	0.90 (0.85–0.94)	0.91 (0.85–0.96)	0.89 (0.85–0.93)	<0.001
*Lifestyle information*				
Any alcohol consumption, n (%)	578 (38)	173 (39.5)	405 (37.4%)	0.4
Smoking, n (%)				
Yes	48 (2.76)	31 (4.4)	17 (1.6%)	<0.001
Physical activity, n (%)				
Moderate	260 (19.7)	49 (17.1)	211 (20.4%)	0.10
High	763 (57.7)	181 (63.3)	582 (56.2%)	
*Dietary patterns*				
Mixed pattern, n (%)	341 (24.7)	286 (94.1)	55 (5.1%)	<0.001
Animal product pattern, n (%)	361 (26.2)	44 (14.5)	317 (29.5%)	<0.001
Roots, tubers and plantain pattern, n (%)	350 (25.4)	21 (6.9)	329 (30.6%)	<0.001
*Laboratory information*				
Albuminuria, n (%)	110 (5.98)	38 (5)	72 (6.6%)	0.2
Triglycerides (mmol/L), median (IQR)	0.86 (0.64–1.16)	0.70 (0.52–0.98)	0.98 (0.74–1.31)	<0.001
High-density lipoprotein (mmol/L), median (IQR)	1.29 (1.08–1.55)	1.45 (1.21–1.71)	1.20 (1.02–1.43)	<0.001
Low-density lipoprotein (mmol/L), median (IQR)	3.01 (2.41–3.71)	3.02 (2.52–3.66)	3.01 (2.32–3.73)	0.5
Cholesterol (mmol/L), median (IQR)	4.84 (4.10–5.55)	4.89 (4.27–5.58)	4.79 (3.95–5.53)	0.003
Uric acid (µmol/L), median (IQR)	294 (245–352)	316 (262–371)	284 (239–340)	<0.001
Urine albumin (mg/L), median (IQR)	4.0 (4.0–5.0)	4.0 (4.0–5.0)	4.0 (4.0–7.3)	0.4
Urine creatinine (mmol/L), median (IQR)	10 (6–15)	10 (7–15)	10 (6–15)	0.5
Albumin creatinine ratio (mg/mmol), median (IQR)	0.53 (0.35–0.85)	0.50 (0.33–0.78)	0.55 (0.36–0.92)	0.002
Estimated glomerular filtration rate, median (IQR)	86 (76–100)	87 (77–100)	86 (75–100)	<0.001
*Underlying conditions*				
Hypertension, n (%)	711 (38.54)	401 (52.6)	310 (28.6%)	<0.001
Diabetes, n (%)	119 (6.45)	71 (9.3)	48 (4.4%)	<0.001
Obesity, n (%)	426 (23.1)	250 (32.9)	176 (16.3%)	<0.001
*Use of medication for the underlying health conditions*				
Hypertension medication, n (%)	267 (14.47)	187 (24.5)	261 (24.1%)	<0.001
Diabetes medication, n (%)	49 (2.66)	38 (5)	11 (1.0%)	<0.001

Data are presented as percentages, means (SDs), or median (interquartile rangeIQR).

Percentages are rounded to one decimal point and may not sum to 100%.

### CKD incidence by geographical location and metabolic factors

**Figure 2:**
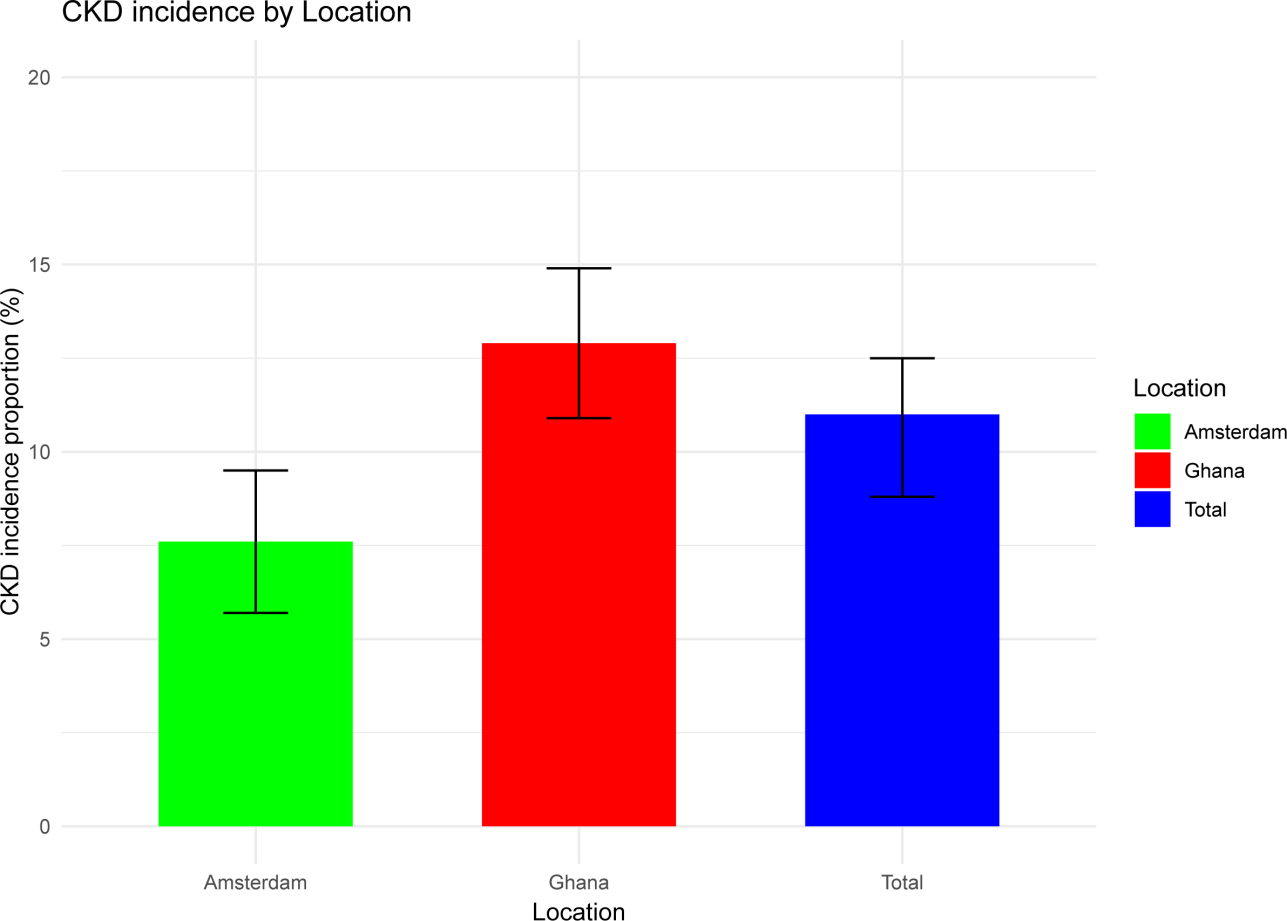
Chronic kidney disease (CKD) incidence by Amsterdam versus Ghana.

**Figure 3:**
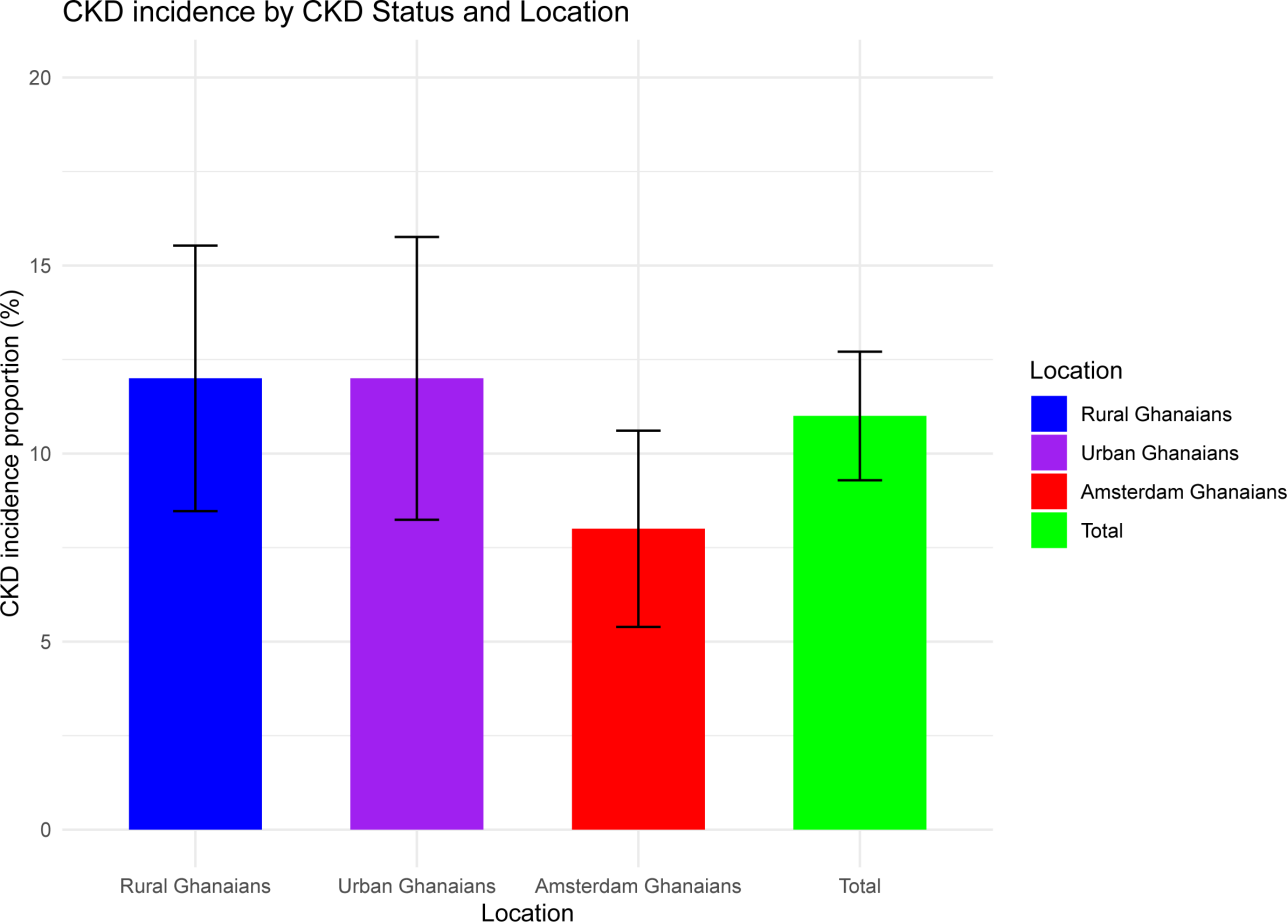
Chronic kidney disease (CKD) incidence by geographical location.

A total of 200 participants developed CKD over the study period. The overall age- and sex-standardised CKD incidence proportion was 11.0% (95% CI 9.3% to 12.7%). CKD incidence proportions were 7.6% (95% CI 5.4% to 10.6%) among Amsterdam Ghanaians and 12.9% (95% CI 10.9% to 14.9%) among Ghanaians residing in Ghana (figure 2). Within Ghana, the CKD incidence was comparable in urban Ghana (12.3%, 95% CI 8.2% to 15.8%) to rural Ghana (12.5%, 95% CI 8.5% to 15.5%) (figure 3).

CKD incidence proportions varied by metabolic factors (online supplemental S1 figure). In general, CKD incidence was higher among those with diabetes, hypertension than those without in Ghana but not in Amsterdam. On the other hand, there were no statistically significant differences in CKD incidence by obesity across all geographical locations (online supplemental S1 figure).

### Predictors of CKD incidence

Among participants who did not have CKD at baseline, we identified age (IRR=1.01, 95% CI 1.01 to 1.03), female sex (IRR=1.65, 95% CI 1.02 to 2.72), uric acid levels (IRR=1.01, 95% CI 1.02 to 1.03), alcohol consumption (IRR=1.45, 95% CI 1.03 to 2.03) and hypertension (IRR=1.63, 95% CI 1.14 to 2.34) as CKD incidence predictors (see table 2). Other variables, including smoking, physical activity, education level, waist-hip ratio, BMI, diabetes, obesity, dietary patterns, TC and triglycerides, did not show significant associations with CKD incidence. Sex and alcohol were further assessed by geographical location due to significant interactions with geographical location (p-values 0.001 and 0.03, respectively; online supplemental S2 table).

**Table 2:** One-step Poisson regression (robust) in participants without CKD at baseline (n=1845)

Variable	Categories	Variance inflation factor score	One-step regressionIncidence rate ratio (95% CI)
General information			
Geographical location	Rural	5.42	1.00 (reference)
	Urban		1.06 (0.66 to 1.69)
	Amsterdam		**0.33(0.13to0.85)**
Age (years)		1.37	**1.01(1.01to1.03)**
Sex	Males	1.91	1.00 (reference)
	Females		**1.65(1.02to2.72)**
Alcohol consumption	No	1.11	1.00 (reference)
	Yes		**1.45(1.03to2.03)**
Smoking	No	1.24	1.00 (reference)
	Yes		0.73 (0.11 to 2.46)
	Past		0.93 (0.43 to 1.79)
Physical activity	Low	1.18	1.00 (reference)
	Moderate		0.93 (0.57 to 1.52)
	High		0.99 (0.67 to 1.50)
Education	Never/elementary	1.35	1.00 (reference)
	Lower secondary		0.68 (0.45 to 1.01)
	Higher secondary		0.76 (0.40 to 1.34)
	Tertiary		0.53 (0.15 to 1.35)
Body mass index (kg/m^2^)		3.13	1.01 (0.96 to 1.06)
Underlying health conditions			
Hypertension	No	1.26	1.00 (reference)
	Yes		**1.63(1.14to2.34)**
Diabetes	No	1.16	1.00 (reference)
	Yes		1.57 (0.87 to 2.67)
Obesity	No	2.48	1.00 (reference)
	Yes		0.98 (0.54 to 1.78)
Dietary patterns			
Mixed pattern		1.25	1.11 (0.75 to 1.58)
Animal product pattern		2.84	0.85 (0.69 to 1.05)
Roots, tubers and plantain pattern		1.73	0.97 (0.80 to 1.16)
Laboratory information			
Uric acid (µmol/L)		1.52	**1.01(1.02to1.03)**
Triglycerides (mmol/L)		1.33	1.01 (0.75 to 1.32)
Total cholesterol (mmol/L)		1.40	0.97 (0.83 to 1.14)

Results obtained via one- step Poisson regression using mfx package in R. CKD incidence (binary outcome) was defined based on the race-free CKD-EPI 2021 equation.

The bold values indicate that they were statistically significant, and the ones not bold indicate they were not statistically significant.

CKDchronic kidney disease

### Association of geographical location with CKD incidence

Among participants without CKD at baseline, significant geographical disparities in CKD incidence emerged. Amsterdam Ghanaians showed notably lower CKD incidence rates than Ghanaians residing in Ghana, even after adjusting for multiple demographic, lifestyle and health factors (aIRR=0.32, 95% CI 0.13 to 0.77). Within Ghana, there were no statistically significant differences in CKD incidence between urban Ghanaians and rural Ghanaians before and after adjustments (aIRR=1.07, 95% CI 0.69 to 1.67; table 3).

**Table 3:** Participants without CKD at baseline: association between geographical location and CKD incidence

Location	n	Model 1IRR (95% CI)	Model 2IRR (95% CI)	Model 3IRR (95% CI)	Model 4IRR (95% CI)
General location comparison					
Ghana	140/1068	1.00 (reference)	1.00 (reference)	1.00 (reference)	1.00 (reference)
Amsterdam	60/757	**0.62(0.45to0.84)**	**0.68(0.49to0.93)**	**0.41(0.17to0.96)**	**0.32(0.13to0.77)**
Site-specific comparison					
Rural Ghana	74/552	1.00 (reference)	1.00 (reference)	1.00 (reference)	1.00 (reference)
Urban Ghana	66/516	1.06 (0.75 to 1.49)	1.12 (0.79 to 1.59)	1.24 (0.81 to 1.88)	1.07 (0.69 to 1.67)
Amsterdam	60/757	**0.64(0.45to0.91)**	0.72 (0.50 to 1.04)	0.48 (0.19 to 1.22)	**0.34(0.13to0.89)**

Results based on robust Poisson regression. location, incidence. CKD is defined based on the race-free CKD-EPI 2021 equation. ratio with confidence interval. Model for age and sex. Model education. Model smoking, physical activity, dietary patterns, alcohol consumption. Model obesity, diabetes, uric acid, hypertension, hypertension and diabetes treatment.

Predictor indicates geographical location. Outcome indicates CKD incidence. CKD is defined based on the race-free CKD-EPI 2021 equation. Model 1 indicates adjusted for age and sex. Model 2 indicates model 1 + education. Model 3 indicates model 2 + smoking, physical activity, dietary patterns and alcohol consumption. Model 4 indicates model 3 + obesity, diabetes, uric acid, hypertension, hypertension and diabetes treatment.

The bold values indicate that they were statistically significant, and the ones not bold indicate they were not statistically significant.

CKDchronic kidney diseaseIRRincidence rate ratio

### Progression to ESKD

Among participants with CKD at baseline (n=258), the majority were women (73%), with a mean age of 52 years (SD=11 years). Amsterdam Ghanaians were younger (50 vs 52 years), had higher education levels and were more likely to work full time (40.8% vs 21.2%). They also had higher obesity rate (31.8% vs 24.1%) and better lipid profiles, with higher HDL and lower triglycerides. Ghanaians residing in Ghana had a more plant-based diet, particularly rich in roots and tubers, and similar hypertension rates (64.8% vs 58.2%; online supplemental S3 table). Within Ghana, urban Ghanaian participants had higher albuminuria prevalence (66.3%), along with higher hypertension (66.3%) and obesity (39.3%) prevalence compared with Amsterdam and rural Ghanaians. Rural Ghanaians showed higher physical activity (58.0%) and diabetes (18.5%) prevalences compared with their urban and Amsterdam counterparts (online supplemental S4 table).

Overall, CKD progression to ESKD was observed among 4 of 258 participants (1.6%, 95% CI 1.0% to 2.1%), with 0.0% in Amsterdam Ghanaians and 2.3% (95% CI 1.3% to 3.2%) among Ghanaians residing in Ghana. Within Ghana, 2.5% (95% CI 1.4% to 3.6%) was in rural Ghanaians and 2.3% (95% CI 1.3% to 3.2%) in urban Ghanaians. Multivariable Poisson regression analysis could not be performed due to these low progression numbers (models did not converge).

### Sensitivity analysis

Overall, most characteristics showed no significant differences between the two groups (online supplemental S5 table). However, we observed some variations in specific aspects. In the rural sample, we found higher representation of participants in the included group than in the participants that were not included. Additionally, physical activity levels, HDL levels and cholesterol were higher in the included group than the group that was not included.

Conversely, for participants who were lost to follow-up, we observed higher levels of urine creatinine, indicating potential differences in kidney function. Moreover, hypertension, diabetes and the use of diabetes medications were more prevalent in the lost to follow-up group.

## Discussion

### Key findings

We aimed to comprehensively assess the incidence, predictors and progression of CKD among rural Ghanaians and urban Ghanaians, as well as among Ghanaian migrants in the Netherlands over a 7-year period. We found that one-tenth of the study population had incident CKD, with Ghanaians residing in Ghana having higher incidence compared with Amsterdam Ghanaians. Age, female sex, alcohol consumption, uric acid levels and hypertension were long-term predictors of CKD incidence. CKD progression to ESKD was minimal.

### Discussion of key findings

Our study found an age- and sex-adjusted CKD incidence of 11% over a 7-year period. This translates to an average annual incidence of 1.6%. To date, there are no population-based longitudinal studies on CKD pertaining to Africans or African migrants in high-income countries. This makes our longitudinal study a pioneering endeavour in these under-represented populations. Our results on age- and sex-adjusted CKD incidence proportions are similar to those reported in clinically based studies among people with diabetes in Ethiopia (10% at both 5- and 10-year follow-ups).^10 11^ The comparable CKD incidence in the general population to people with diabetes in Ethiopia is disturbing, implying the increasing nature of the disease even in those without mainstream risk factors. On the other hand, our age- and sex-adjusted CKD incidence proportions fall in the middle of population estimates from around the world. For instance, Framingham Offspring study in the USA found a lower rate, with only 7.9% developing CKD over an 18.5-year period, averaging an annual estimate of 0.4%.^22^ Similar to our findings, a 10-year follow-up of a community-based cohort of 123 764 Japanese adults found that 16% (19 802) developed CKD, resulting in an annual estimate of 1.6%.^23^ On the other hand, Cardiovascular Health Study in the USA observed incidence proportions of 16.9% (approximately 1.9% annually) among participants aged 45 or older over a 9-year follow-up higher than our study.^24^ It is worth noting that the definition of CKD varied across studies. It is possible that each method for the study was chosen based on accuracy in that study population as accuracy of CKD methods varies across populations.^22^,^24^ Despite these variations, the observed incidences underscore the pressing need for CKD interventions across the globe.

Although we hypothesised that higher prevalence of CKD risk factors like hypertension and diabetes among Ghanaian migrants in Amsterdam would result in a greater CKD incidence compared with rural and urban Ghana, we identified higher incidence rates among rural and urban Ghanaians (12% for both) compared with Amsterdam Ghanaians (8%). This pattern is consistent with the pattern from our previous prevalence study, which showed higher CKD prevalence among Ghanaians living in Ghana compared with their migrant peers in Europe (13.7% in urban Ghanaians, 12.6% in rural Ghanaians and 9.1% in Amsterdam Ghanaians).^7^ These consistent results suggest that CKD prevalence and incidences are similarly influenced by geographical context.

One significant factor that may contribute to the differences in CKD incidence between Ghana and the Netherlands is access to healthcare and treatment for CKD risk factors like hypertension and diabetes. Although we accounted for diabetes and hypertension treatment, it is possible that variations in the types, quality and effectiveness of these treatments could still be contributing to the observed disparities. For example, according to WHO’s 2020 data, 97% of people in Europe had access to basic healthcare services, whereas only 52% of people in Africa had similar access.^25^ Future studies should thoroughly investigate in detail how access to healthcare influences CKD within various settings.

We identified age, female sex, hypertension, alcohol consumption and uric acid levels as long-term predictors of CKD incidence, which could additionally help explain geographical differences observed. Ageing has long been associated with reduced kidney function due to decline in nephron count and blood flow.^26^ Hypertension is known for its harmful impact on kidney microvasculature and nephrons, disrupting filtration process and causing proteinuria.^27^ Hormonal factors, such as those related to menopause, may contribute to sex differences in CKD.^28^ Excessive alcohol consumption can dehydrate and strain kidneys, further increasing CKD risk.^29^ Elevated uric acid levels can lead to kidney damage through crystal formation.^30^ Beyond its role in crystal formation, high uric acid levels may impair nitric oxide synthesis, a critical factor in vascular health, leading to decreased renal perfusion and worsening kidney function over time. While these factors varied depending on geographical context (table 1), their inclusion in our models only offered a partial explanation for CKD incidence proportions across geographical locations. Notably, adjusting for these factors did not lead to significant changes in the results, indicating the prominent role of the other plausible factors.

Another plausible factor that could be contributing to disparities in CKD incidence between Ghana and the Netherlands is the prevalence of infections such as hepatitis B, hepatitis C, complicated malaria and schistosomiasis, which have been linked to CKD yet more commonly found in Africa compared with Europe.^31^ Additionally, recent evidence suggests that APOL1 risk alleles, which increase CKD risk among African ancestry populations, may be more likely to initiate kidney injury among those exposed to chronic viral infections.^32^ Furthermore, the higher utilisation of herbal medications for various reasons in the African context may contribute to the observed disparity in CKD rates between Ghana and the Netherlands.^33^ Herbal medicines can harm the kidneys through various mechanisms, such as the presence of nephrotoxic compounds. While infectious diseases and herbal medications are plausible contributors to the CKD incidence disparities observed, we could not account for them in our study and further research is warranted.

We observed low CKD progression rates to ESKD among participants with CKD at baseline. Although our sample size was too small to conduct detailed statistical modelling, the low rates of progression to ESKD we observed are noteworthy, especially when compared with findings in other migrant populations. For instance, rapid CKD progression to ESKD among South Asian migrants has been reported as 40 times faster (higher chance) compared with their European counterparts.^34^ Given the unexpected nature of our findings, further investigation with larger sample sizes is warranted to elucidate the factors contributing to this phenomenon.

This study has significant clinical and public health implications as it reveals a concerning trend in CKD incidence. The fact that CKD incidence is high raises concern, emphasising the need for immediate public health interventions. First, our study identifies rural and urban areas in Ghana as hotspots for CKD incidence where intervention efforts should be encouraged. To develop these interventions, it is crucial to first understand specific factors driving CKD incidence in this context, such as infectious diseases, herbal medicine use and access to healthcare. Second, screening for long-term predictors such as uric acid levels and hypertension may help to identify at-risk individuals. Third, identifying factors associated with CKD progression is crucial for effective disease management which can inform better healthcare strategies.

### Strength and limitations

Our study has several strengths. Being the first population-based longitudinal study to explore incidence, predictors and progression within the same group across different contexts enables feasible comparisons. The inclusion of three distinct geographical locations (rural, urban and Amsterdam) enhances our understanding of epidemiological transitions driven by urbanisation and migration. Nonetheless, certain limitations warrant acknowledgement. In our study, sex was identified as a predictor of CKD incidence, necessitating separate analyses by sex. However, we had already stratified by geographical location and comorbidities, and we also conducted long-term predictor, progression and sensitivity analyses. Stratifying by sex would significantly reduce the sample sizes in most of the analyses, making it impossible to complete the comprehensive paper. Nonetheless, a deeper understanding of why women are at greater risk of CKD in this population is warranted, requiring studies with larger sample sizes. Our prospective cohort study had a significant attrition rate of 52%, primarily due to the impact of the COVID-19 pandemic. Lockdowns and fear of infection, especially in urban Ghana where all follow-up data collection occurred during the pandemic, deterred some participants from participating in the follow-up. While sociodemographic and lifestyle factors demonstrated were similar between included and participants lost to follow-up, the variations we found in specific factors like urine creatine and cholesterols and comorbidities suggest the need for cautious interpretation when generalising our results to the entire RODAM population. We also recognise that some participants lost to follow-up might have progressed to ESKD or even died, but we did not have data to assess whether deaths from CKD was the reason for loss to follow-up. Furthermore, we acknowledge that multiple regression models could not be fit for CKD progression, which limited our ability to explore the role of key risk factors, such as diabetes and hypertension, in CKD progression. Unfortunately, our study lacked data on medicinal and herbal treatments, APOL1 genetic variations, as well as on infectious diseases, valuable insights given the relevance of these factors within Africa, beyond metabolic risks. Furthermore, the absence of iohexol and cystatin C data, a recommended CKD measurement tool for Africans and the general population, is noteworthy. If creatinine already underestimates the risk of CKD, then the potential implications with iohexol and cystatin C (potentially an even higher incidence rate) are even more concerning, further underscoring the urgency of addressing CKD as a public concern.

## Conclusion

One-tenth of Ghanaians developed CKD over 7 years, with higher incidence in Ghana compared with Europe. Age, female sex, alcohol use, uric acid levels and hypertension were predictive factors. CKD progression to ESKD was minimal. High CKD incidence among Ghanaians, especially non-migrants, calls for in-depth assessment of contributing factors and targeted intervention.

## supplementary material

10.1136/bmjgh-2024-016786 online supplemental file 1

## Data Availability

Data are available upon reasonable request.
