# Supplementary material for: Incidence, long-term predictors and progression of chronic kidney disease among African migrants and non-migrants: the transcontinental population-based prospective RODAM cohort study
Source: BMJ Glob Health. 2025 Jan 20;10(1):e016786. doi: 10.1136/bmjgh-2024-016786 (PMC11749268; doi:10.1136/bmjgh-2024-016786)
Supplement: online supplemental file 1 [file bmjgh-10-1-s001.pdf]

S1 Table: In participants without CKD at baseline: Baseline characteristics by geographical location.

Variables	Total n=1845	Amsterdam n= 762	Urban n= 526	Rural n=557	p-value
Demographics					
**Follow-up time (years), mean (SD)**	6.7 (0.67)	6.78 (0.90)	6.68 (0.47)	6.64 (0.38)	<0.001
**Age, mean (SD)**	46 (10.9)	46 (9.6)	44 (10.5)	47 (12.8)	<0.001
**Sex, n (%)**					
Females	1170 (63.4)	460 (60.4)	366 (69.6)	344 (61.8)	<0.001
Males	675 (36.6)	302 (39.6)	160 (30.4)	213 (38.2)	
**Education, n (%)**					
Never/Elementary	719 (41.0)	217 (30.5)	186 (36.3)	316 (59.7)	<0.001
Lower secondary	666 (38.0)	264 (37.1)	236 (46.1)	166 (31.4)	
High secondary	274 (15.6)	177 (24.9)	62 (12.1)	35 (6.6)	
Tertiary	94 (5.4)	54 (7.6)	28 (5.5)	12 (2.3)	
**Employment status, n (%)**					
Full time	445 (32.2)	172 (46.2)	142 (28.5)	131 (25.6)	<0.001
Part-time	739 (53.5)	89 (23.9)	304 (61.0)	346 (67.6)	
Social benefits	71 (5.1)	69 (18.5)	2 (0.4)	0 (0.0)	
Retired	15 (1.1)	1 (0.3)	9 (1.8)	5 (1.0)	
Full time homemaker	17 (1.2)	6 (1.6)	6 (1.2)	5 (1.0)	
Unable to work	83 (6.0)	30 (8.1)	29 (5.8)	24 (4.7)	
Student	12 (0.9)	5 (1.3)	6 (1.2)	1 (0.2)	
Anthropometry information					
**BMI (kg/m^2), median (IQR)**	26.0 (22.4-29.7)	28.0 (25.2-30.8)	26.9 (23.6-30.7)	21.9 (19.6-25.1)	<0.001
**Waist hip ratio, median (IQR)**	0.90 (0.85-0.94)	0.91 (0.85-0.96)	0.90 (0.86-0.94)	0.89 (0.85-0.93)	<0.001
Lifestyle information					
**Any alcohol consumption, n (%)**	578 (38)	173 (39.5)	168 (31.9)	237 (42.5)	<0.001
**Smoking, n (%)**					
Yes	48 (2.76)	31 (4.4)	5 (1.0)	12 (2.3)	<0.001
Past	118 (6.78)	59 (8.4)	24 (4.7)	35 (6.6)	
**Physical activity, n (%)**					
Moderate	260 (19.7)	49 (17.1)	86 (16.9)	125 (23.8)	<0.001
High	763 (57.7)	181 (63.3)	267 (52.4)	315 (59.9)	
Dietary patterns					
**Mixed pattern, n (%)**	341 (24.7)	286 (94.1)	16 (3.1)	39 (7.0)	<0.001
**Animal product pattern, n (%)**	361 (26.2)	44 (14.5)	206 (39.6)	111 (20.0)	<0.001
**Roots, tubers, and plantain pattern, n (%)**	350 (25.4)	21 (6.9)	57 (11.0)	272 (49.1)	<0.001
Laboratory information					
**Albuminuria, n (%)**	110 (5.98)	38 (5)	48 (9.1)	24 (4.3)	<0.001
**Triglycerides (mmol/L), median (IQR)**	0.86 (0.64-1.16)	0.70 (0.52-0.98)	1.01 (0.75-1.34)	0.95 (0.74-1.22)	<0.001
**HDL (mmol/L), median (IQR)**	1.29 (1.08-1.55)	1.45 (1.21-1.71)	1.23 (1.06-1.45)	1.17 (0.97-1.40)	<0.001

**LDL (mmol/L), median (IQR)**	3.01 (2.41-3.71)	3.02 (2.52-3.66)	3.43 (2.74-4.06)	2.59 (2.08-3.28)	<0.001
**Cholesterol(mmol/L), median (IQR)**	4.84 (4.10-5.55)	4.89 (4.27-5.58)	5.19 (4.49-5.92)	4.26 (3.61-3.28)	<0.001
**Uric acid (μmol/L), median (IQR)**	294 (245-352)	316 (262-371)	286 (237-340)	284 (242-339)	<0.001
**Urine albumin(mg/L), median (IQR)**	4.0 (4.0-5.0)	4.0 (4.0-5.0)	4.0 (4.0-9.2)	4.0 (4.0-4.0)	<0.001
**Urine creatinine(mmol/L), median (IQR)**	10 (6-15)	10 (7-15)	11 (8-17)	8 (5-13)	<0.001
**Albumin creatinine ratio(mg/mmol), median(IQR)**	0.53 (0.35-0.85)	0.50 (0.33-0.78)	0.50 (0.33-0.85)	0.59 (0.39-0.95)	<0.001
**eGFR, median (IQR)**	86 (76-100)	87 (77-100)	82 (73-95)	90 (77-103)	<0.001
Underlying conditions					
**Hypertension, n (%)**	711 (38.54)	401 (52.6)	168 (31.9)	142 (25.5)	<0.001
**Diabetes, n (%)**	119 (6.45)	71 (9.3)	31 (5.9)	17 (3.1)	<0.001
**Obesity, n (%)**	426 (23.1)	250 (32.9)	147 (27.9)	29 (5.2)	<0.001
Use of medication for the underlying health conditions					
**Hypertension medication, n (%)**	267 (14.47)	187 (24.5)	42 (8)	38 (6.8)	<0.001
**Diabetes medication, n (%)**	49 (2.66)	38 (5)	9 (1.7)	2 (0.4)	<0.001

Data are presented as percentages, means (SDs), or median (interquartile range). Percentages are rounded to one decimal point and may not sum to 100%. % = Percentages. eGFR= Estimated glomerular filtration rate. BMI=body mass index. HDL=High density lipoprotein cholesterol, LDL=Low density lipoprotein cholesterol.

S1 Figure: Incidence proportion by Geographical location and metabolic factors

CKD incidence by diabetes status and geographical location

Age- and sex-standardised CKD incidence proportions were higher among those with diabetes compared to those without in rural and urban Ghana but not Amsterdam. The CKD proportions for those with diabetes were rural 19.8% (95%CI, 16.2-23.6), urban-Ghanaians 28.4% (95%CI, 23.7-32.0) and Amsterdam-Ghanaians 10.0% (95%CI, 6.1-14.0). On the other hand, among those without diabetes, CKD incidence proportions were: 7.6% in Amsterdam-Ghanaians (95%CI, 5.8-9.4), 9.6% in urban-Ghanaians (95%CI, 7.1-11.0) and 12.4% in Rural-Ghanaians (95%CI, 9.9-14.9)

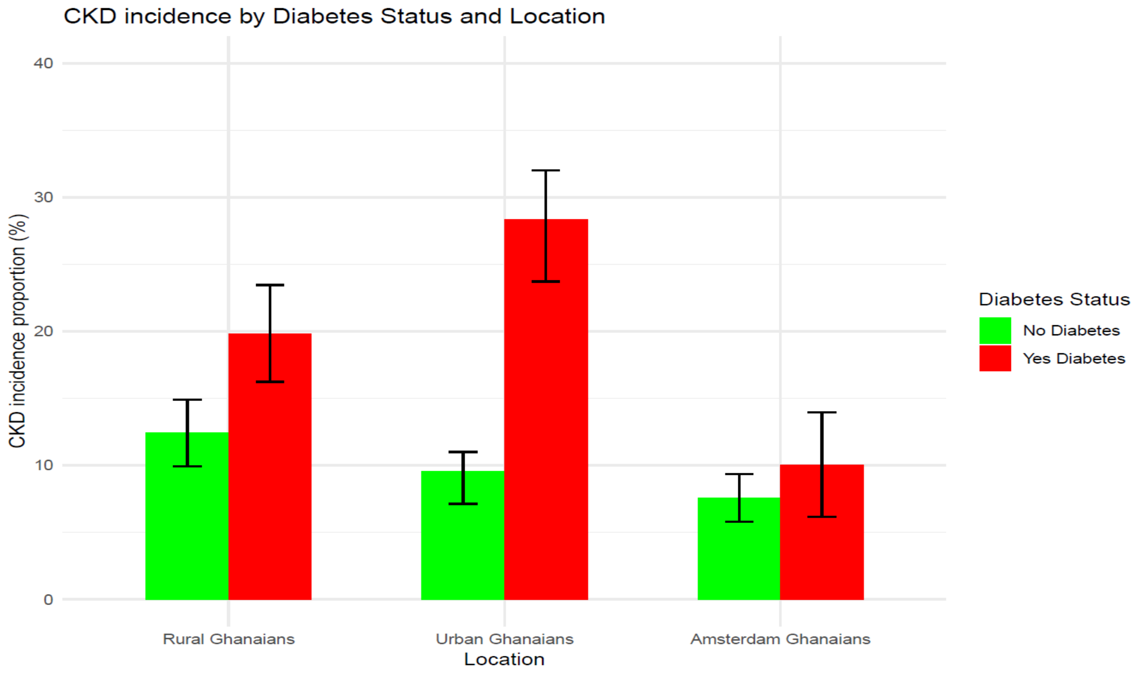

Age-sex adjusted CKD incidence by diabetes status and geographical location. Error bars indicate 95% confidence intervals.

CKD incidence by hypertension status and geographical location

A similar pattern was observed for age- and sex-standardised CKD incidence proportions among Ghanaians with hypertension, mirroring the diabetes status across geographical location. CKD incidence proportions among those living in urban-Ghana was 16.7% (95%CI, 9.7-15.4), 18.7% (95%CI, 15.5-23.0) in rural-Ghana, and 8.7% (95%CI, 6.4-11.0) among Amsterdam-Ghanaians. Ghanaians without hypertension had CKD incidence of 6.2% (95%CI, 3.7-8.7) in urban-Ghana, 9.8% (95%CI, 6.8-12.7) in rural-Ghana, and 7.1% (95%CI, 4.4-9.8) in Ghanaians residing in Amsterdam.

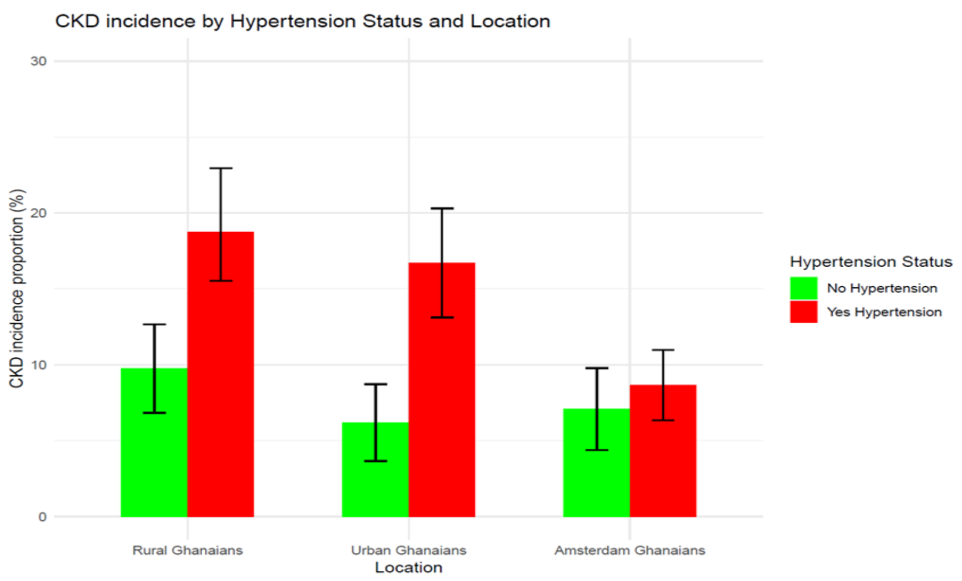

Age-sex adjusted CKD incidence by hypertension status and geographical location. Error bars indicate 95% confidence intervals.

CKD incidence by obesity status and geographical location

In contrast to hypertension and diabetes status, age- and sex-standardised CKD incidence proportions between those with or without obesity did not vary significantly (with a slightly higher incidence in non-obese rural residents than obese rural residents). Among Ghanaians with obesity living in urban-Ghana, CKD incidence was 12.5% (95%CI, 9.7-15.4), 10.0% (95%CI, 7.2-12.8) in rural-Ghana, and 8.9% (95%CI, 6.0-11.7) among Amsterdam-Ghanaians. For Ghanaians without obesity, CKD incidence was 11.9% (95%CI, 8.8-15.1) in urban-Ghana, 13.2% (95%CI, 10.6-15.9) in rural-Ghana, and 8.3% (95%CI, 5.9-10.7) in Amsterdam-Ghanaians.

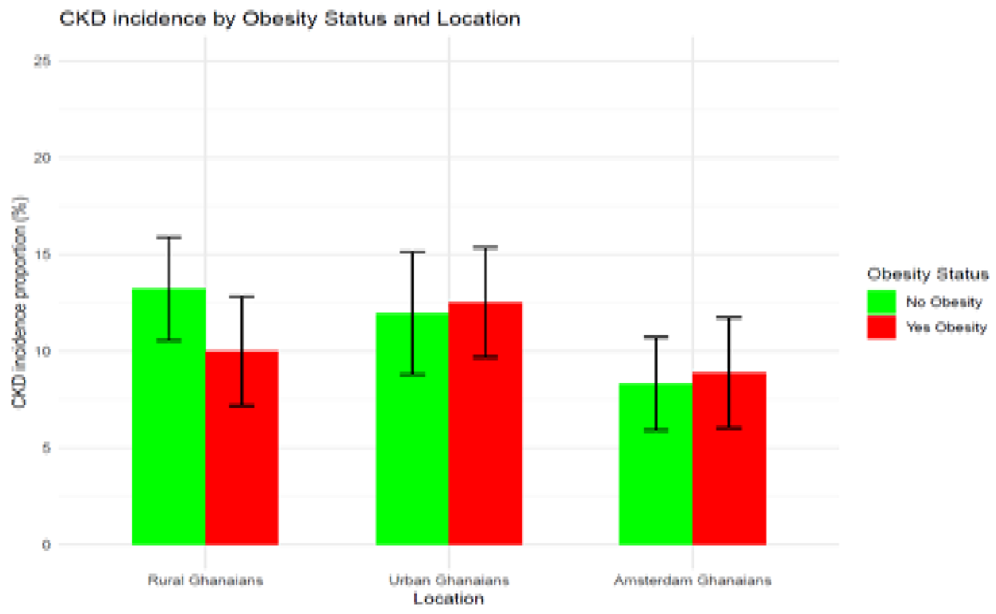

Age-sex adjusted CKD incidence by obesity status and geographical location. Error bars indicate 95% confidence interval.

S2 Table: Association of sex and alcohol consumption with CKD incidence by geographical location

Sex and alcohol were further assessed by geographical location due to significant interactions with geographical location. Females in rural Ghana had two times the risk of CKD than males in rural Ghana. There were no statistically significant associations between sex and CKD incidence in Amsterdam and in urban Ghana. Any alcohol consumption in urban Ghana had nearly two times the risk of CKD than no consumption at all in urban Ghana. There were no statistically significant associations between any alcohol consumption and CKD incidence in Amsterdam and in rural Ghana.

**Variables**	**N= 1845**	**Amsterdam**<br><br>**IRR (95% CI)**	**Urban Ghana**<br><br>**IRR (95% CI)**	**Rural Ghana**<br><br>**IRR (95% CI)**	**p-value**
**Sex**					
Males	675	1.00 (Reference)	1.00 (Reference)	1.00 (Reference)	0.001
Females	1170	0.65 (0.39-1.09)	1.16 (0.69-2.05)	**2.07 (1.23-3.67)**	
**Alcohol consumption**					
No	943	1.00 (Reference)	1.00 (Reference)	1.00 (Reference)	0.030
Yes	578	1.13 (0.55-2.25)	**1.78 (1.09-2.90)**	0.91 (0.57-1.45)	

Association of CKD incidence by sex and alcohol consumption by geographical location

S3 Table: In participants with CKD at baseline: Baseline characteristics of Amsterdam Vs Ghana

Variables	Total<br>n=258	Amsterdam<br>n= 88	Ghana<br>n= 170	p-value
Demographics				
**Follow-up time, mean (SD)**	6.7 (0.62)	6.8 (0.81)	6.68 (0.47)	<0.001
**Age, mean (SD)**	52 (11.18)	50 (8.53)	52 (12)	<0.001
**Sex, n (%)**				
**Females**	189 (73.3)	55 (62.5)	134 (78.8%)	0.005
**Males**	69 (26.3)	33 (37.5)	36 (21.2%)	
**Education, n (%)**				
**Never/Elementary**	119 (49.8)	28 934.1)	91 (58.0%)	0.002
**Lower secondary**	80 (33.5)	33 (40.2)	47 (29.9%)	
**Higher secondary**	35 (14.6)	19 (23.2)	16 (10.2%)	
**Tertiary**	5 (2.1)	2 (2.4)	3 (1.9%)	
**Employment status, n (%)**				
**Full time**	53 (25.9)	20 (40.8)	33 (21.2%)	<0.001
**Part-time**	111 (54.1)	12 (24.5)	99 (63.5%)	
**Social benefits**	9 (4.4)	7 (14.3)	2 (1.3%)	
**Retired**	8 (3.9)	2 (4.1)	6 (3.8%)	
**Full time homemaker**	1 (0.5)	1 (2.0)	0 (0.0%)	
**Unable to work**	23 (11.2)	7 (14.30	16 (10.3%)	
Anthropometry information				
**BMI (kg/m²), median (IQR)**	26.73 (23.17- 30.46)	28.23 (25.13-31.43)	25.7 (22.1-29.6)	<0.001
**Waist hip ratio, median (IQR)**	0.92(0.88-0.96)	0.93 (0.88-0.99)	0.92 (0.88-0.95)	0.11
Lifestyle information				
**Any alcohol consumption, n (%)**	71 (30.87)	30 (50)	41 (24.1%)	<0.001
**Smoking, n (%)**				
**Yes**	6 (2.5)	4 (5.0)	2 (1.3%)	0.3
**Past**	15 (6.4)	5 (6.3)	10 (6.4%)	
**Physical activity, n (%)**				
**Moderate**	35 (17.4)	12 (26.7)	23 (14.7%)	0.086
**High**	105 (52.2)	24 (53.3)	81 (51.9%)	
Dietary patterns				
**Mixed pattern, n (%)**	51 (23.8)	42 (91.3)	9 (17.6%)	<0.001
**Animal product pattern, n (%)**	39 (18.2)	7 (15.2)	32 (82.1%)	0.7
**Roots, tubers, and plantain pattern, n (%)**	44 (20.6)	1 (2.2)	43 (97.7%	<0.001
Laboratory information				
**Albuminuria, n (%)**	143 (56.08)	47 (55.3)	96 (56.5%)	0.9
**Triglycerides (mmol/L), median (IQR)**	0.98 (0.70-1.50)	0.74 (0.61-1.08)	1.11 (0.80-1.40)	<0.001
**HDL (mmol/L), median (IQR)**	1.24 (1.04-1.50)	1.46 (1.24-1.75)	1.16 (0.94-1.35)	<0.001
**LDL (mmol/L), median (IQR)**	3.08 (2.58-3.87)	3.02 (2.58-3.71)	3.11 (2.60-3.89)	0.4
**Cholesterol(mmol/L), median (IQR)**	4.90 (4.32-5.64)	5.00 (4.48-5.67)	4.84 (4.16-5.64)	0.3

**Uric acid (µmol/L), median (IQR)**	300 (255-373)	368 (285-450)	286 (254-349)	<0.001
**Urine albumin(mg/L), median (IQR)**	25 (4-61)	24 (4-65)	25 (4-58)	0.7
**Urine creatinine(mmol/L), median (IQR)**	10 (6-14)	9 (5-15)	10 (6-14)	0.6
**Albumin creatinine ratio(mg/mmol), median (IQR)**	3 (1-6)	3 (1-6)	3 (1-6)	0.7
**eGFR, median (IQR)**	68 (57-86)	62 (57-87)	69 (57-86)	0.4
Underlying conditions				
**Hypertension, n (%)**	156 (60.47)	57 (64.8)	99 (58.2%)	0.3
**Diabetes, n (%)**	46 (17.83)	16 (18.2)	30 (17.6%)	>0.9
**Obesity, n (%)**	69 (26.74)	28 (31.8)	41 (24.1%)	0.2
Use of medication for the underlying health conditions				
**Hypertension medication, n (%)**	67 (25.97)	32 (36.4)	35 (20.6%)	0.006
**Diabetes medication, n (%)**	18 (6.98)	8 (9.1)	10 (5.9%)	0.3

Data is presented as percentages, means (SDs), or median (25th, 75th percentiles). Percentages are rounded to one decimal point and may not sum to 100%. Median (IQR)=median (interquartile range). % = Percentages. eGFR= estimated glomerular filtration rate. BMI=body mass index. HDL=High density lipoprotein cholesterol, LDL=Low density lipoprotein cholesterol. Level of education: Higher vocational=higher vocational schooling or university. Intermediate=Intermediate vocational schooling or intermediate/higher secondary schooling. Lower vocational=Lower vocational schooling or lower secondary schooling. Never/elementary=Never been to school or elementary schooling.

S4 Table: In participants with CKD at baseline: Baseline characteristics

Variables	Total n=258	Amsterdam n= 88	Urban Ghana n= 89	Rural Ghana n=81	p-value
Demographics					
**Follow-up time, mean (SD)**	6.7 (0.62)	6.8 (0.81)	6.7 (0.52)	6.6 (0.42)	<0.001
**Age, mean (SD)**	52 (11.18)	50 (8.53)	52 (10.22)	53 (14.22)	<0.001
**Sex, n (%)**					
Females	189 (73.3)	55 (62.5)	70 (78.7)	64 (79.0)	<0.001
Males	69 (26.3)	33 (37.5)	19 (21.3)	17 (21.0)	
**Education, n (%)**					
Never/Elementary	119 (49.8)	28 934.1)	41 (46.6)	50 (72.5)	<0.001
Lower secondary	80 (33.5)	33 (40.2)	31 (35.2)	16 (23.2)	
Higher secondary	35 (14.6)	19 (23.2)	14 (15.9)	2 (2.90	
Tertiary	5 (2.1)	2 (2.4)	2 (2.3)	1 (1.4)	
**Employment status, n (%)**					
Full time	53 (25.9)	20 (40.8)	16 (18.40	17 (24.6)	<0.001
Part-time	111 (54.1)	12 (24.5)	59 (67.8)	40 (58.0)	
Social benefits	9 (4.4)	7 (14.3)	2 (2.3)	0 (0.0)	
Retired	8 (3.9)	2 (4.1)	5 (5.7)	1 (1.4)	
Full time homemaker	1 (0.5)	1 (2.0)	0 (0.0)	0 (0.0)	
Unable to work	23 (11.2)	7 (14.30	5 (5.7)	11 (15.9)	
Anthropometry information					
**BMI (kg/m^2), median (IQR)**	26.73 (23.17-	28.23 (25.13-31.43)	28.74 (25.70-	22.67 (20.54-	<0.001
**Waist hip ratio, median (IQR)**	0.92(0.88-0.96)	0.93 (0.88-0.99)	0.92 (0.89-0.95)	0.90 (0.85-0.96)	<0.001
Lifestyle information					
**Any alcohol consumption, n**	71 (30.87)	30 (50)	16 (18)	25 (30.9)	<0.001
**Smoking, n (%)**					
Yes	6 (2.5)	4 (5.0)	0 (0.0)	2 (2.9)	<0.001
Past	15 (6.4)	5 (6.3)	6 (6.8)	4 (5.9)	
**Physical activity, n (%)**					
Moderate	35 (17.4)	12 (26.7)	12 (13.8)	11 (15.9)	<0.001
High	105 (52.2)	24 (53.3)	41 (47.1)	40 (58.0)	
Dietary patterns					
**Mixed pattern, n (%)**	51 (23.8)	42 (91.3)	3 (3.4)	6 (7.5)	<0.001
**Animal product pattern, n (%)**	39 (18.2)	7 (15.2)	22 (25.0)	10 (12.5)	<0.001
**Roots, tubers, and plantain**	44 (20.6)	1 (2.2)	12 (13.6)	31 (38.8)	<0.001
Laboratory information					
**Albuminuria, n (%)**	143 (56.08)	47 (55.3)	59 (66.3)	37 (45.7)	<0.001
**Triglycerides (mmol/L), median**	0.98 (0.70-1.50)	0.74 (0.61-1.08)	1.11 (0.81-1.36)	1.11 (0.80-1.42)	<0.001
**HDL (mmol/L), median (IQR)**	1.24 (1.04-1.50)	1.46 (1.24-1.75)	1.20 (1.02-1.42)	1.12 (0.90-1.29)	<0.001
**LDL (mmol/L), median (IQR)**	3.08 (2.58-3.87)	3.02 (2.58-3.71)	3.52 (2.86-4.26)	2.87 (2.26-3.44)	<0.001
**Cholesterol(mmol/L), median**	4.90 (4.32-5.64)	5.00 (4.48-5.67)	5.35 (4.60-6.04)	4.46 (3.89-5.05)	<0.001

**Uric acid (μmol/L), median**	300 (255-373)	368 (285-450)	288 (255-355)	282 (251-332)	<0.001
**Urine albumin(mg/L), median**	25 (4-61)	24 (4-65)	29 (11-58)	18 (4-58)	<0.001
**Urine creatinine(mmol/L),**	10 (6-14)	9 (5-15)	12 (7-17)	8 (5-12)	<0.001
**Albumin creatinine ratio(mg/mmol), median (IQR)**	3 (1-6)	3 (1-6)	3 (1-6)	3 (1-5)	<0.001
**eGFR, median (IQR)**	68 (57-86)	62 (57-87)	67 (57-85)	72 (57-89)	<0.001
Underlying conditions					
**Hypertension, n (%)**	156 (60.47)	57 (64.8)	59 (66.3)	40 (49.4)	<0.001
**Diabetes, n (%)**	46 (17.83)	16 (18.2)	15 (16.9)	15 (18.5)	<0.001
**Obesity, n (%)**	69 (26.74)	28 (31.8)	35 (39.3)	6 (7.4)	<0.001
Use of medication for the underlying health conditions					
**Hypertension medication, n (%)**	67 (25.97)	32 (36.4)	25 (28.1)	10 (12.3)	<0.001
**Diabetes medication, n (%)**	18 (6.98)	8 (9.1)	7 (7.9)	3 (3.7)	<0.001

Data is presented as percentages, means (SDs), or median (25th, 75th percentiles). Percentages are rounded to one decimal point and may not sum to 100%. Median (IQR)=median (interquartile range). % = Percentages. eGFR= estimated glomerular filtration rate. BMI=body mass index. HDL=High density lipoprotein cholesterol, LDL=Low density lipoprotein cholesterol. Level of education: Higher vocational=higher vocational schooling or university. Intermediate=Intermediate vocational schooling or intermediate/higher secondary schooling. Lower vocational=Lower vocational schooling or lower secondary schooling. Never/elementary=Never been to school or elementary schooling.

S5 Table: Comparison of characteristics of participants included and not included in the study.

**Variables**	**Participants Included N=2183**	**Participants Lost to Follow Up N=2390**	**P-value for differences between groups**
Location, n (%)			
Rural Ghana	649(29.7)	462 (19.3)	<0.001
Urban Ghana	615 (28.2)	840 (35.1)	
Amsterdam	919 (42.1)	1088 (45.5)	
Demographic information			
**Age, mean (SD)**	46.24 (11.33)	45.78 (12.62)	0.194
**Sex, (n (%))**			
Males	772(35.4)	863(36.1)	0.621
Females	1411 (64.6)	1527(63.9)	
**Education, n (%)**			
Tertiary	101 (4.9)	119 (5.4)	0.246
Higher secondary	320 (15.6)	358 (16.1)	
Lower secondary	772 (37.6)	770 (34.7)	
Never/Elementary	859 (41.9)	974 (43.9)	
**Employment status, n (%)**			
Employed full time	516 (31.7)	678 (33.3)	<0.001
Employed part-time	865 (53.2)	925 (45.4)	
Full time home maker	18 (1.1)	19 (0.9)	
On social benefits	84 (5.2)	144 (7.1)	
Retired	23 (1.4)	48 (2.4)	
Student	15 (0.9)	40 (2.0)	
Unable to work	106 (6.5)	185 (9.1)	
Anthropometry information			
**BMI (kg/m2), medium(IQR)**	26.08 (22.44-29.79)	26.12 (22.48-30.09)	0.380
**Waist hip ration, (mean (SD))**	0.90 (0.07)	0.90 (0.07)	0.552
Lifestyle information			
**Any alcohol consumption, n (%)**	659 (36.8)	809 (34.9)	0.219
**Smoking, n (%)**			
Yes	59 (2.9)	56 (2.6)	0.24
Past	135 (6.6)	173 (7.9)	
**Physical activity, n (%)**			
Moderate	299 (19.3)	354 (18.8)	<0.001
High	888 (57.2)	984 (52.4)	
Dietary patterns			
**Mixed pattern, n (%)**	404 (25.0)	473 (25.0)	<0.001
**Animal product pattern, n (%)**	404 (25.0)	473 (25.0)	<0.001
**Roots, tubers, and plantain pattern, n (%)**	404 (25.0)	473 (25.0)	<0.001
Laboratory information			
**Albuminuria, n (%)**	254 (12.0)	329 (14.2)	0.036
**Triglycerides (mmol/L), (mean (SD))**	0.95 (0.63)	0.94 (0.68)	0.592
**HDL (mmol/L), (mean (SD))**	1.28 (0.52)	1.20 (0.58)	<0.001

**LDL (mmol/L), (mean (SD))**	3.00 (1.12)	3.02 (1.31)	0.575
**Cholesterol(mmol/L), (mean (SD))**	4.86 (1.13)	4.71 (1.67)	<0.001
**Uric acid (µmol/L), (mean (SD))**	295.96 (90.95)	298.46 (105.92)	0.420
**Urine albumin(mg/L), (mean (SD))**	16.12 (79.05)	20.68 (98.90)	0.088
**Urine creatinine(mmol/L), (mean (SD))**	11.26 (7.20)	11.75 (7.45)	0.027
**Albumin creatinine ratio(mg/mmol), (mean (SD))**	1.52 (5.99)	1.97 (8.86)	0.048
**eGFR, (mean (SD))**	86.19 (17.19)	86.06 (18.66)	0.807
Underlying conditions			
**Hypertension, n (%)**	887 (41.2)	1072 (44.9)	0.016
**Diabetes, n (%)**	165 (7.6)	232 (9.7)	0.019
**Obesity, n (%)**	507 (23.6)	608 (25.5)	0.145
Use of medication for the underlying health conditions			
**Hypertension medication, n (%)**	791 (36.8)	939 (39.3)	0.089
**Diabetes medication, n (%)**	67 (3.1)	104 (4.4)	0.035

Data is presented as percentages, n (%)) and (means (SDs)). Percentages are rounded to one decimal point and may not sum to 100%. % = Percentages. eGFR= Estimated glomerular filtration rate. BMI=body mass index. HDL=High density lipoprotein cholesterol, LDL=Low density lipoprotein cholesterol. Level of education: Higher vocational=higher vocational schooling or university. Intermediate=Intermediate vocational schooling or intermediate/higher secondary schooling. Lower vocational=Lower vocational schooling or lower secondary schooling. Never/elementary=Never been to school or elementary schooling only.
